# A nanodiamond-formulated plant protein induces robust immunity against porcine epidemic diarrhea virus in piglets

**DOI:** 10.3389/fimmu.2025.1674222

**Published:** 2025-09-19

**Authors:** Thuong Thi Ho, Hoai Thu Tran, Phuong Minh Thi Nguyen, Huyen Thi Bui, Hien Thu Thi Nguyen, Thao Bich Thi Le, Minh Dinh Pham, Wesley Wei-Wen Hsiao, Dai Huu Nguyen, Ha Hoang Chu, Ngoc Bich Pham, Hang Thu Thi Hoang

**Affiliations:** ^1^ Institute of Biology, Vietnam Academy of Science and Technology, Ha Noi, Vietnam; ^2^ Department of Chemical Engineering, National Taiwan University of Science and Technology, Taipei, Taiwan; ^3^ CNC Veterinary Medicine Trading and Production Joint Stock Company,, Ha Noi, Vietnam; ^4^ Graduate University of Science and Technology, Vietnam Academy of Science and Technology, Ha Noi, Vietnam

**Keywords:** nanodiamond, COE-S1D-pII protein, PEDV, immune response, vaccine, piglets

## Abstract

Porcine epidemic diarrhea virus (PEDV) continues to be a major infectious threat in swine, especially endangering piglets. The COE and S1D domains have been identified as crucial antigens suitable for designing subunit vaccines. Nanodiamonds (NDs), owing to their biocompatibility, large surface area, and modifiable surfaces, have gained interest as novel carriers to improve recombinant protein vaccines. In this study, we transiently expressed a COE-S1D fusion protein containing the GCN4pII motif (COE-S1D-pII) in *Nicotiana benthamiana*. The recombinant protein was subsequently mixed with nanodiamonds at various mass ratios to form COE-S1D:ND complexes. SDS-PAGE and Western blot analyses identified the optimal ratio as 1:24 (w/w). Additional size, zeta and morphology characterization of these complexes was carried out. We then assessed the immune response of the COE-S1D:ND complex (1:24, w/w) in pregnant sows and their piglets, comparing it to the response induced by the free COE-S1D-pII protein. After administering a booster dose, the COE-S1D:ND mixture significantly enhanced PEDV-specific IgG and COE-S1D-specific IgA levels, as well as neutralizing antibody titers, as measured by ELISA and virus neutralization assays in their piglets. Overall, the results highlight that ND nanoparticles can strengthen both systemic and mucosal immunity, supporting the potential of using plant-produced COE-S1D-pII protein in combination with nanodiamonds as a next-generation subunit vaccine candidate against PEDV.

## Introduction

Porcine epidemic diarrhea virus (PEDV) is an RNA virus, which is classified within the genus Alphacoronavirus of the Coronaviridae family (1). PEDV is a primary viral agent responsible for inducing severe watery diarrhea in pigs (2). Although PEDV is capable of infecting pigs at any age, it is particularly devastating in suckling piglets, where it can cause acute enteric disease with mortality rates as high as 80–100% (3). Porcine epidemic diarrhea (PED) thus remains a significant infectious threat to swine production worldwide, especially in Asia, where repeated outbreaks continue to result in considerable economic losses (4, 5). The classical genogroup (G1) PEDV strains are generally associated with mild or low-pathogenic infections, whereas the more virulent genogroup 2 (G2) strains—including subgroups G2a and G2b—have been linked to outbreaks causing mortality rates approaching 100% in suckling piglets (6). In Vietnam, large-scale PEDV outbreaks have largely been driven by G2 strains (7). Despite ongoing efforts, a vaccine that effectively protects immunologically naïve pigs against PEDV is still lacking, underscoring the continued challenge of managing PED outbreaks (8).

Among the four structural proteins of PEDV, the Spike (S) protein is crucial for facilitating the virus’s entry into host cells and serves as the principal immunogen, inducing neutralizing antibody responses (9). Owing to these critical functions, the S protein is considered the primary target for subunit vaccine design. Structurally, it comprises two distinct domains: the S1 domain (amino acids 1–789) and the S2 domain (amino acids 790–1383) (10). Within the S1 domain, two major neutralizing epitopes have been identified: the COE epitope (amino acids 499–638) and a recently characterized region, S1D (amino acids 636–789). These epitopes have been shown to stimulate strong neutralizing antibody responses against PEDV, making them attractive candidates for the development of next-generation subunit vaccines (11, 12).

To date, the COE and S1D proteins of PEDV have been successfully expressed as fusion proteins with various motifs in multiple heterologous systems, including *Escherichia coli* (13), yeast (14), mammalian cells (15), and plants (16). Among these platforms, plant-based expression systems—particularly transient expression via agroinfiltration—have emerged as promising strategies owing to their rapid production, scalability, and cost-effectiveness (17, 18). In our previous studies, COE protein variants were transiently expressed in *N. benthamiana* as fusion constructs with the GCN4pII motif (pII), and their immunogenic potential was demonstrated in animal models (19, 20). Notably, the COE protein derived from a highly virulent Vietnamese PEDV G2a strain, when fused with the pII motif (designated COE/G2a-pII), elicited protective immune responses in piglets born to vaccinated pregnant sows against PEDV G2a strain (20). Nevertheless, the neutralizing antibody titers detected in these piglets remain suboptimal and need to be improved to ensure stronger and broader protection, especially against the predominant PEDV G2b strains circulating in Vietnam. Although antibodies produced in sera alone are not enough for complete protection against PEDV infection, the levels of neutralizing antibodies in the serum have been associated with enhanced resistance to PEDV (21). This highlights the importance of investigating alternative antigens or antigen combinations that can trigger stronger neutralizing responses to achieve broader protection. In this regard, the extended COE-S1D fragment—which includes both the COE region and the adjacent S1D neutralizing epitope—offers the potential to expand antigenic coverage. To date, no studies have reported the transient expression of the COE-S1D protein (amino acids 499–789) fused with the pII motif in plants, nor its immunogenicity in animal models. To further improve the immunogenicity of these plant-produced COE-S1D-pII proteins, we investigated the potential of incorporating nanodiamonds (ND) as novel nanoparticle-based adjuvants and delivery vehicles.

In designing modern protein subunit vaccines that direct immune responses toward defined epitopes, nanoparticles have proven invaluable. Their widespread application as antigen and drug carriers, or as adjuvants, stems from their ability to reduce side effects, increase formulation stability, and vigorously promote humoral immune responses (22, 23). NDs combine several appealing features, such as excellent biocompatibility, easily modifiable surface chemistry, a high capacity to carry antigens, and lower toxicity compared to other carbon nanomaterials (24–27). In addition, incorporating nanoparticles as vaccine adjuvants has been shown to enhance immune responses (28, 29).

In this study, we produced COE-S1D protein in *N. benthammiana* via transient expression. We aimed to explore whether incorporating NDs could enhance the immunogenicity of plant-produced COE-S1D-pII proteins against PEDV. We assessed immune responses in pregnant sows and their piglets to better represent the target host context. By comparing the immune responses elicited by the COE-S1D-pII: ND formulation (abbreviated as COE-S1D:ND) with those induced by the free plant-based COE-S1D-pII proteins, we aimed to determine the adjuvant potential of nanodiamonds. Overall, our results show that the COE-S1D:ND complex elicits markedly stronger immune responses against a highly virulent PEDV G2b strain than the free COE-S1D protein. This underscores the promise of using nanodiamonds as nanoparticle-based adjuvants in the development of subunit vaccines for PEDV.

## Method

### Production and characterization of plant-based COE-S1D-pII protein of PEDV

The coding sequence corresponding to amino acids 499–789 of the S protein of PEDV strain NAVET/PEDV/PS6/2010 (genotype G2a) was selected to generate the COE-S1D construct. The nucleotide sequence was codon-optimized for efficient expression in *N. benthamiana*. The optimized fragment was fused in-frame to a series of functional elements, including: (i) an N-terminal 6×His tag to enable affinity purification and detection; (ii) the GCN4pII motif to facilitate trimerization, and (iii) the KDEL signal for protein accumulation in the endoplasmic reticulum. This synthetic expression cassette was driven by the Cauliflower mosaic virus (CaMV) 35S promoter and inserted into the pRTRA vector backbone. The assembled cassette was subsequently subcloned into the binary vector pCB301 for *Agrobacterium tumefaciens*–mediated transient expression in *N. benthamiana* via vacuum infiltration, following the procedure described previously (19).

At five days post-agroinfiltration, the infiltrated *N. benthamiana* leaf tissues were harvested and immediately frozen at –80 °C for downstream processing. Expression of the COE-S1D-pII protein in *N. benthamiana* leaves was detected and semi-quantified by SDS-PAGE and Western blot. Blots were probed with either a monoclonal anti-His tag antibody or porcine sera from pigs immunized with the commercial PEDV AJ1102 strain vaccine (Corning, Wuhan Keqian Biology) as primary antibodies, followed by goat anti-mouse IgG-HRP (Invitrogen) or goat anti-porcine IgG (H+L)-HRP (SouthernBiotech) as secondary antibodies. Signals were visualized and quantified by comparing band intensities against a standard curve generated from H5N1-specific ScFv protein (30), using ImageQuant TL 8.0 software (Cytiva) and an Amersham™ Imager 680.

Recombinant COE-S1D-pII protein was extracted and purified from the plant biomass by immobilized metal affinity chromatography (IMAC) and size exclusion chromatography (SEC), as previously described (20). The purified protein fraction was formulated in 50% (v/v) glycerol diluted in 1× PBS (pH 7.4) and kept at –20 °C to maintain structural stability and antigenicity. In addition, the multimeric state of the purified COE-S1D-pII protein was evaluated by chemical cross-linking using BS3 (Bis[sulfosuccinimidyl] suberate) (31). Briefly, one µg of COE-S1D-pII protein was reacted with 5 mM BS3 for 30 min at room temperature, then quenched by adding Tris-HCl buffer (pH 8.0) to 50 mM. The products were separated by 4–10% SDS-PAGE under reducing conditions and detected by Western blot using an anti-his tag antibody.

The tertiary structure of the COE-S1D protein was modeled using SWISS-MODEL (32), based on a template selected by sequence similarity. Model quality was evaluated by the GMQE and QMEAN scores provided by the server.

### Preparation of surface-oxidized NDs

To introduce oxygen-containing functional groups, commercial diamond powder (Diamond Innovations, USA) was oxidized by treating it with a 3:1 (v/v) mixture of concentrated sulfuric and nitric acids (H_2_SO_4_:HNO_3_). The oxidation reaction was performed using a Model Discover microwave system (CEM) at approximately 100 °C and 100 W for 3 hours, following the procedure reported (28). After the reaction, residual acids were carefully diluted before collecting the nanodiamonds. All procedures were conducted in a chemical fume hood to minimize exposure to nitrogen dioxide (NO_2_). Residual acids were carefully diluted before collecting the NDs.

### Preparation and characterization of COE-S1D:ND complexes

To generate a uniform ND suspension, surface-oxidized nanodiamonds (NDs) were ultrasonically dispersed in deionized water at a concentration of 10 mg/mL. The COE-S1D-pII protein was then added to the ND suspension at mass ratios of 1:6, 1:12, 1:24, 1:36, and 1:48 in phosphate-buffered saline (1× PBS, pH 7.4). To enhance protein adsorption onto the ND surface, the mixtures were gently sonicated on ice for 5 minutes. Following incubation, samples were spun at 13,000 rpm for 15 minutes. The pellets were gently rinsed twice with deionized water to remove unbound proteins and subsequently resuspended in PBS for further analysis. Protein binding efficiency was determined using the Bradford assay, and complex stability was assessed over a period of one week by monitoring particle size and retained protein content.

The complexes were characterized by SDS-PAGE and Western blot analysis, following the protocol described previously (33). Samples were combined with 4× SDS-PAGE loading buffer and denatured by heating at 95 °C for 20 minutes. After electrophoresis on a 12% SDS-PAGE gel, proteins were transferred to PVDF membranes at 35 V overnight. Membranes were kept in 5% milk in PBS (pH 7.4) for two hours, incubated with anti−6×His tag antibody (Invitrogen) for two hours, then with goat anti−mouse IgG−HRP (Invitrogen) for one hour. Bands were visualized using DAB (Sigma) in 0.05 M Tris−HCl (pH 7.2). Signal detection and quantification were performed with Amersham™ Imager 680, ImageQuant TL 8.0 (Cytiva), and ImageJ.

The COE-S1D:ND complexes were characterized for particle size distribution, polydispersity index (PDI), and zeta potential following the protocol described previously (33). A final concentration of each sample of 50 μg/mL was obtained by diluting in deionized water and measured in triplicate at 37 °C. Hydrodynamic diameter measurements were evaluated using a Zetasizer Nano ZS (Malvern Panalytical), and zeta potential was determined with a Horiba SZ-100 analyzer. Morphological characterization by Transmission Electron Microscopy (TEM) was performed using a JEOL 1400 Flash microscope operating at 120 kV to verify the structural integrity.

### Pig immunization

The animal experiments were approved by the Ethics Committee of the Institute of Biology, Vietnam Academy of Science and Technology (VAST), Hanoi. All procedures conformed to the principles of the “3Rs” and complied with Directive 86/609/EEC of the European Communities Council on the protection and use of laboratory animals. Pigs were housed and closely monitored by veterinarians to minimize stress, pain, and discomfort during the experiments. Two weeks prior to immunization, blood samples were collected from pregnant sows for testing of PEDV-IgG, IgA-specific antibodies, and neutralizing antibodies against PEDV. Six pregnant sows lacking PEDV-IgG, IgA antibodies, and neutralizing antibodies were enrolled. Purified COE-S1D-pII protein (150 µg/dose), the COE-S1D:ND mixture (containing 150 µg COE-S1D protein and 3.6 mg ND per dose), or PBS mixed with ND (3.6 mg/dose) were emulsified with Emulsigen^®^-D adjuvant (MVP) at an 8:2 ratio. At approximately 80 days of gestation, sows were intramuscularly immunized in the neck on days 0 and 14. Blood samples from sows were obtained at day 0 and day 35 after immunization, while milk samples were obtained on day 35 post-immunization. Piglets (n = 5) born to the immunized sows or the PBS: ND control sow were allowed to suckle and were kept with their dams. Blood samples from all piglets were obtained at 5 days of age for further analysis. All sera and milk were kept at −20 °C until measurement of PEDV-specific IgG and IgA antibody responses by ELISA and determination of PEDV-neutralizing antibody titers.

### Evaluation of PEDV-specific IgG antibodies by ELISAs

Serum samples from sows and piglet sera were tested for PEDV-specific IgG using a commercial ELISA kit (INgezim PEDV 11.PED.K.1/5, Eurofins INGENASA). Piglet sera were diluted 1:100 prior to testing. The S/P (sample-to-positive) ratio was calculated as follows: (sample OD − negative control OD)/(positive control OD − negative control OD). Samples were considered positive for PEDV-specific IgG if the S/P value exceeded 0.35.

### Evaluation of COE-S1D-specific IgA antibodies by ELISAs

Levels of COE-specific IgA in sow milk and piglet sera were measured using an indirect ELISA following the previous protocol described (30), with slight adjustments. In brief, SEC-purified COE-S1D protein (100 ng per well, at 1 ng/µL) was used to coat 96-well plates overnight at 4 °C in PBS (pH 7.4). Plates were then incubated in 5% skim milk solution in PBS. To detect IgA, milk and serum samples were applied at serial dilutions ranging from 1:10 to 1:640. The plates were then incubated at room temperature for two hours, washed three times with PBS containing 0.05% Tween 20, and subsequently treated with HRP-conjugated goat anti-mouse IgA (SouthernBiotech) diluted 1:5000 in blocking buffer. After 15 min incubation with TMB substrate, the reaction was stopped by adding 1 M H_2_SO_4_. Absorbance was measured at 450 nm.

### Virus-neutralizing antibody assay

To evaluate neutralizing antibodies in pig sera, a virus neutralization assay was performed based on the method described in (33), with some modifications. The serum samples were heat-inactivated at 56 °C for 30 minutes, followed by preparation of serial dilutions. Each dilution was combined with 10² TCID_50_/0.1 mL of a highly virulent Vietnamese G2b strain of PEDV and incubated at 37 °C for an hour to allow antibody–virus interaction. The mixtures were then added to confluent Vero cell monolayers and left for another hour at 37 °C before being gently washed with PBS to remove unbound virus. After incubation, the cell cultures received fresh α-MEM medium containing trypsin and were kept at 37 °C in a CO_2_ incubator for a period of six days. The neutralizing antibody titer was assessed as the greatest dilution of serum that completely blocked cytopathic effects from appearing in the cell monolayer.

### Statistical analysis

Data analysis was performed using the Mann–Whitney test in GraphPad Prism version 8.0. Results are reported as mean values with their corresponding standard deviations (SD). Statistical significance between groups was accepted at p-values below 0.05. Significance levels are indicated as follows: *p < 0.05; **p < 0.01; ***p < 0.001; **** p < 0.0001.

## Results

### Production and characterization of plant-based COE-S1D-pII protein of PEDV

**Figure 1:**
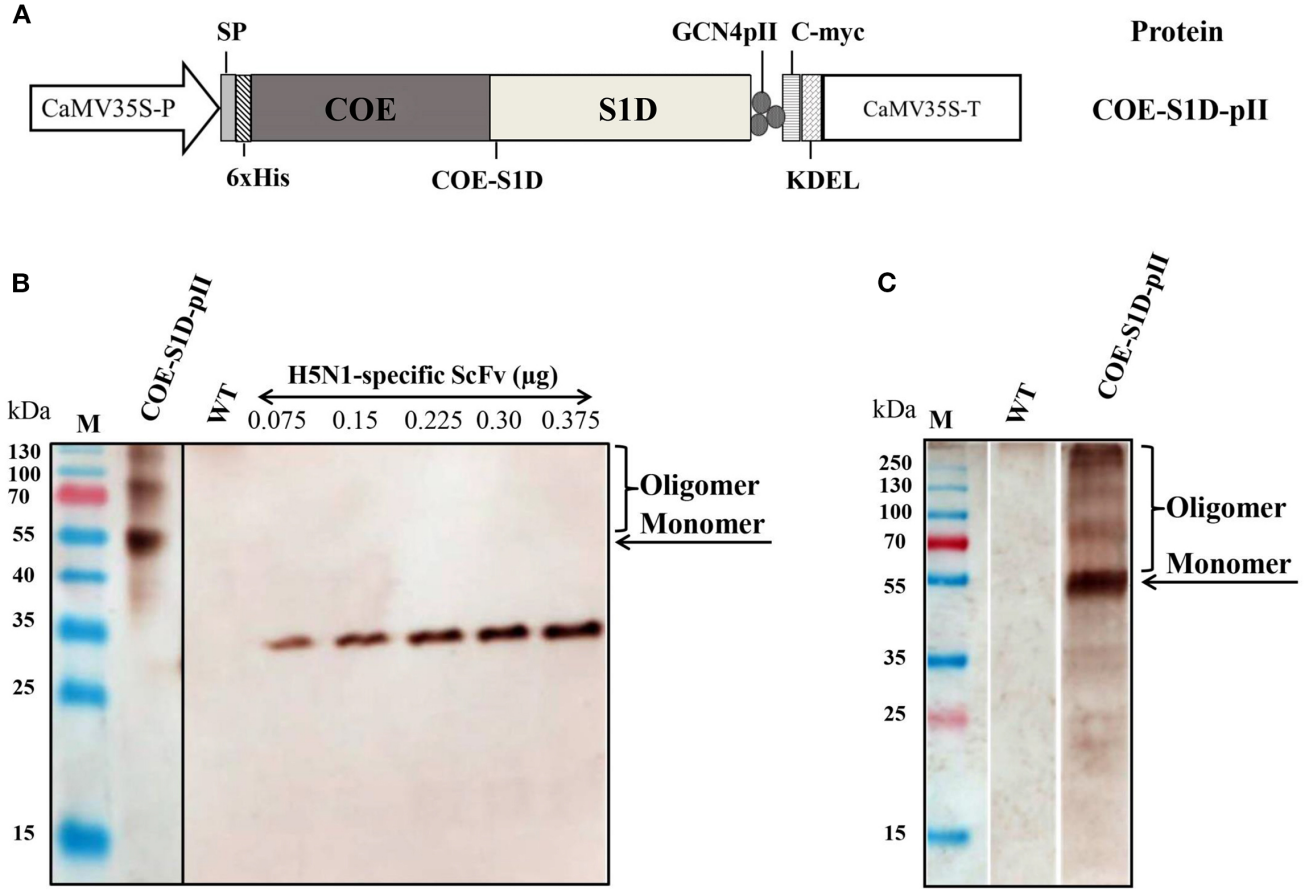
Construction of the expression vector and transient expression of COE-S1D-pII protein in *N. benthamiana*. **(A)** The plant expression cassette carries a DNA sequence that encodes COE-S1D fused with the pII motif. The components of the cassette include: the Cauliflower mosaic virus (CaMV) 35 S promoter (CaMV35S-P), LeB4 signal peptide (SP); 6x His tag (6xHis); the GCN4pII motif (pII); c-myc tag (c-myc); endoplasmic reticulum retention signal (KDEL), the CaMV 35 S terminator (CaMV35S-T) **(B)**. The COE-S1D-pII protein, expressed in *N. benthamiana* leaves, is detected through Western blotting. WT: Wild type *N. benthamiana*. This detection employs anti-His monoclonal antibody and HRP-conjugated goat anti-mouse IgG antibody. H5N1-specific ScFv antibodies were utilized to establish a standard curve. The accumulation of COE-S1D protein in leaves was then calculated using ImageQuant TL (Cytiva) after capture by the Amersham™ Imager 680 (Cytiva) **(C)**. The COE-S1D protein is also detected in *N. benthamiana* leaves using anti-PEDV polyclonal antibodies and HRP-conjugated anti-pig IgG as primary and secondary antibodies, respectively.

To achieve the expression of the COE-S1D-pII protein in *N. benthamiana*, a plant-based expression vector harboring the target gene was constructed (**Figure 1A**). This recombinant vector was subsequently introduced into *A. tumefaciens* through transformation, enabling the delivery of the expression cassette into the plant system. The accumulation of the COE-S1D-pII protein in *N. benthamiana* tobacco leaves was detected via Western blot analysis using an anti-His antibody. SDS-PAGE and Western blot results confirmed the successful expression of the COE-S1D-pII antigen in tobacco leaves, as evidenced by a band on the membrane with an apparent molecular weight greater than 55 kDa. The theoretical molecular weight of the recombinant monomeric COE-S1D-pII antigen is calculated to be 38.2 kDa. However, the experimentally observed molecular weight of the monomeric COE-S1D antigen was found to be greater than 55 kDa. The difference between the theoretical and observed molecular weights of the COE-S1D-pII antigen may be attributed to glycosylation modifications, which can affect antigen migration and separation during SDS-PAGE. In silico analysis using NetNGlyc 1.0 identified six predicted N-linked glycosylation sites in the COE-S1D region. Such N-glycans are expected to increase the apparent molecular weight and retard electrophoretic migration during SDS-PAGE. This interpretation is consistent with published data showing that the PEDV spike is N-glycosylated (33–35). Importantly, biochemically that PNGase F (but not O-glycosidase) increased the electrophoretic mobility of recombinant PEDV S1, confirming predominant N-linked glycans and their effect on apparent molecular mass (36). Together, these findings, plus our prediction of six N-glycosylation sites in COE-S1D-pII, might provide a mechanistic explanation for the discrepancy between theoretical and observed molecular weights. The COE-S1D-pII antigen was not detected in the leaf extracts of non-transgenic *N. benthamiana* plants. The accumulation level of the recombinant COE-S1D antigen in *N. benthamiana* leaves was semi-quantitatively analyzed using Western blot. The concentration and signal intensity of specific ScFv H5 bands (27) were used to construct a standard curve with ImageQuant TL software (Cytiva) after image acquisition with an Amersham™ Imager 680 (Cytiva). The semi-quantitative analysis revealed that the COE-S1D protein accumulated in tobacco leaves at approximately 115 mg/kg of fresh leaves, accounting for 1.95% of the total soluble protein (**Figure 1B**). This accumulation level is comparable to that of the COE protein (20).

Furthermore, the expression of the recombinant COE-S1D-pII protein in *N. benthamiana* leaves was also detected using pig sera immunized with the Corning (Wuhan Keqian Biology) vaccine containing the PEDV AJ1102 strain (**Figure 1C**). The Western blot results in **Figure 1C** showed a band on the membrane with a molecular weight consistent with the theoretical calculation, approximately 55 kDa. This finding suggests that the COE-S1D-pII protein produced in *N. benthamiana* leaves retains antigenic properties similar to those of the natural PEDV antigen.

### Purification and characterization of the oligomeric state of the COE-S1D-pII protein

**Figure 2:**
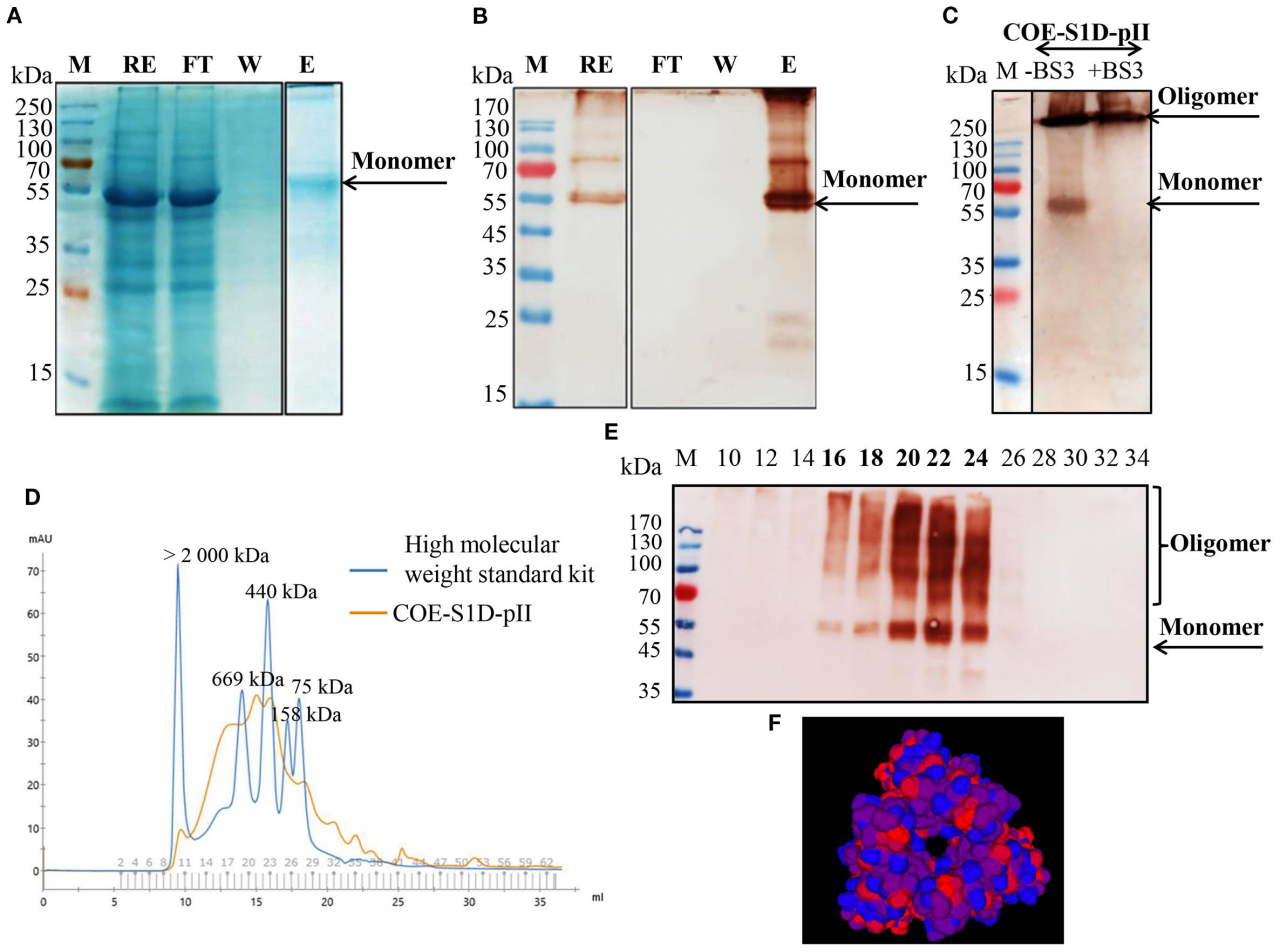
Purification and characterization of the multimeric state of the COE-S1D-pII protein. **(A, B)** Purification of COE-S1D-pII protein by IMAC. Raw extract (RE), flow-through (FT), Wash (W), and elute (E) were separated on SDS-PAGE, then stained in Coomassie staining solution, and washed in destaining solution (A). Raw extract (RE), flow-through (FT), wash (W), and elute (E) were separated on SDS-PAGE, then blotted and detected using a monoclonal anti-c-myc antibody **(C)** The oligomeric states of COE-S1D were investigated by performing cross-linking reactions. COE-S1D-pII protein was mixed with (+) or without (-) BS3 cross-linkers for 0 mM and 5 mM concentrations, respectively. The mixture was visualized via SDS-PAGE and immunoblotting with an anti-His antibody. **(D)** Size exclusion chromatography (SEC) profiling of COE-S1D-pII protein. A standard kit for molecular weight estimation (75–2000 kDa, GE) was used. **(E)** COE-S1D-pII Protein in SEC fractions was identified through Western blot analysis. **(F)** The tertiary structure of the COE-S1D-pII protein was predicted using the SWISS-MODEL homology modeling server. Red regions are strongly hydrophobic, blue regions are strongly hydrophilic, and purple regions are intermediate, containing mixed or moderately hydrophobic residues.

COE-S1D-pII protein was firstly purified by IMAC. The presence of the COE-S1D-pII protein in fractions after IMAC purification was analyzed using SDS-PAGE, followed by Coomassie Blue staining and further confirmation through Western blot analysis (**Figures 2A, B**). The results indicated that the COE-S1D-pII protein was absent in the flow-through (FT) and wash (W) fractions. The purified COE-S1D-pII antigen was predominantly detected in the elution (E) fraction.

The IMAC-purified COE-S1D-pII protein was subjected to SEC and cross-linking analysis to assess its oligomeric characteristics. The oligomeric state of the COE-S1D protein was assessed using BS3 cross-linking. SDS-PAGE analysis combined with Coomassie Blue staining and Western blotting showed a band larger than 250 kDa when BS3 was present (**Figure 2C**). Along with the results from SEC analysis, this suggests that the COE-S1D protein predominantly forms oligomers under natural conditions.

In addition, the SEC purification results, along with Western blot analysis indicated that the COE-S1D-pII protein was detected in fractions 16–24, corresponding to a molecular weight range of approximately 440–669 kDa (**Figures 2D, E**). The tertiary structure of the COE-S1D-pII protein was constructed using SWISS-MODEL, based on the cryo-electron microscopy (cryo-EM) structure of the PEDV S glycoprotein (PDB ID: 6VV5.1.A) as a template (**Figure 2F**). This template shares a sequence identity of 95.65% with the COE-S1D protein, supporting the accuracy of the model. The predicted model yielded a GMQE score of 0.75 and a QMEANDisCo Global score of 0.80 ± 0.05, indicating overall sound quality and reliability of the structure for downstream analyses. The resulting model predicted that the COE-S1D-pII protein forms a homo-trimer, consistent with the trimeric organization of the native PEDV spike glycoprotein.

### Characterization of the COE-S1:ND complexes

**Figure 3:**
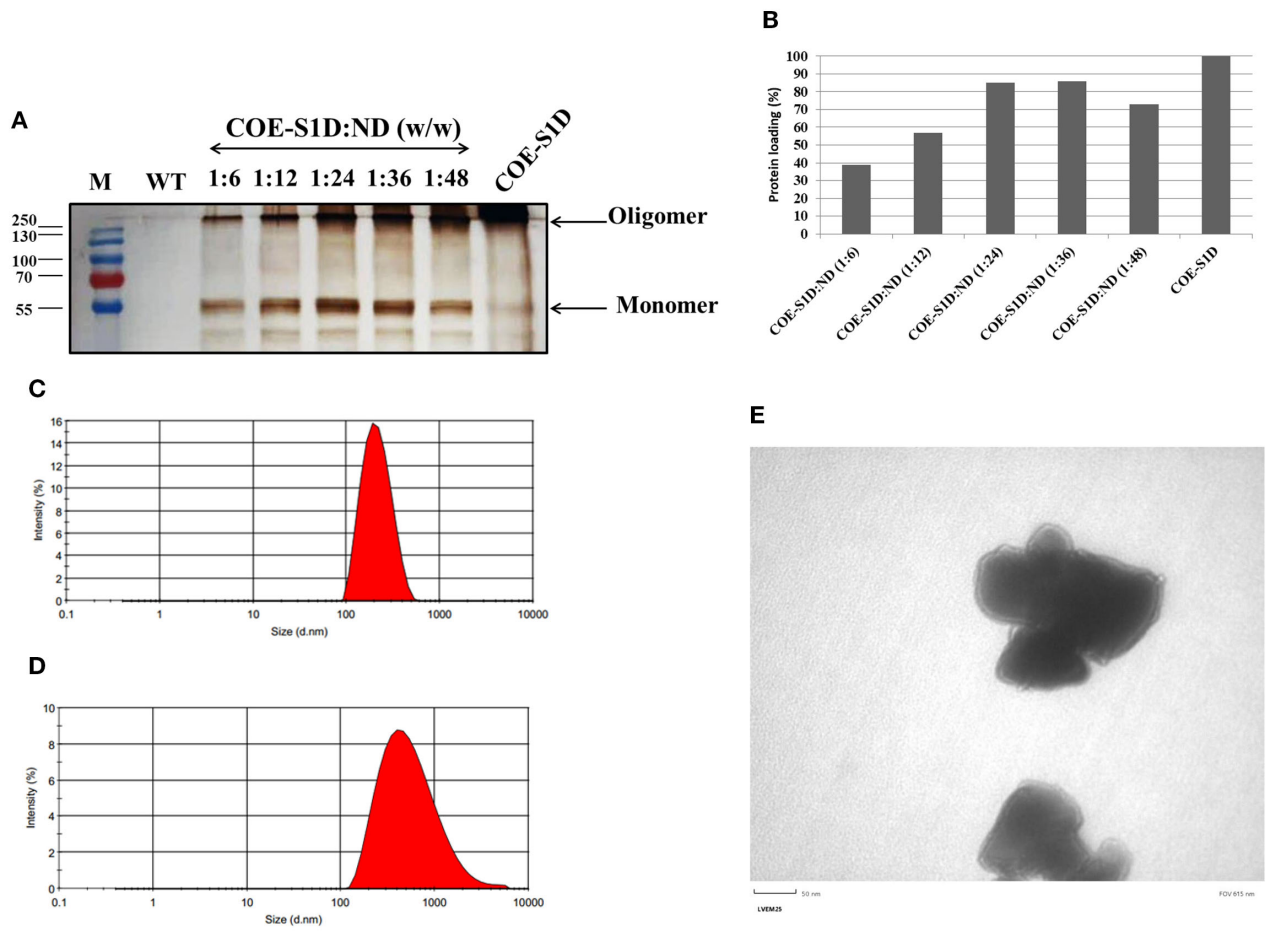
Optimization and characterization of the COE−S1D:ND complexes. **(A)** COE-S1D protein (500 ng/µL) was mixed with ND at different mass ratios (1:6–1:48, w/w) in 1X PBS (pH 7.4) and sonicated for 5 min at 4°C. After centrifugation, particles were washed twice with deionized water and resuspended in 1X PBS (pH 7.4). Free and complexed COE-S1D:ND were analyzed by 12% SDS−PAGE, transferred to membranes, and detected using anti−His tag antibody and HRP−conjugated secondary antibody; **(B)** The percentage of COE-S1D-pII protein bound to ND at different mixing ratios was quantified using ImageJ software based on Western blot images. Hydrodynamic size distribution profiles of ND nanoparticles **(C)**, and the COE:S1D:ND complexes (1:24, w/w) **(D)** dispersed in water. **(E)** TEM images of the COE-S1D:ND complexes (1:24, w/w) at a magnification of 50 nm.

The COE-S1:ND complexes were prepared by sonicating physical mixtures of COE-S1D-pII protein and ND particles at varying mass ratios (1:6, 1:12, 1:24, 1:36, and 1:48). To identify the ratio that provided the highest binding efficiency of COE-S1D-pII protein to the ND surface, Bradford assay, SDS-PAGE, and Western blot analyses were performed (**Figures 3A, B**). Results from the Bradford assay and Western blot confirmed successful adsorption of COE protein onto the ND. Analysis using ImageJ software revealed that mixing COE-S1D-pII protein with ND at mass ratios of 1:6, 1:12, and 1:24 (w/w) resulted in an increase in protein adsorption efficiency from 39% to 57% and then to 85.3%, respectively. However, further increasing the amount of ND to ratios of 1:36 and 1:48 (w/w) did not significantly improve adsorption levels. Consequently, the 1:24 (w/w) ratio was selected for subsequent experiments.

The hydrodynamic diameter of the ND displayed a mean hydrodynamic diameter of 200.2 nm, while the COE-S1D:ND complexes measured slightly larger at 433.6 nm (**Figures 3C, D**). After coating ND particles with COE-S1D-pII protein (pI 5.6, negatively charged at pH 7.4), the zeta potential of complexes increased from –77.5 mV to –48.2 mV (**Table 1**). This shift might indicate adsorption of positively charged residues (e.g., Lys and Arg) onto the ND surface, partially neutralizing its surface charge, while the complex remained negatively charged overall due to the net negative charge of the protein. Alterations in both particle size and zeta potential were observed the COE-S1D:ND complexes suggests that the adsorption of COE-S1D-pII protein onto the surface of ND.

**Table 1:** Physicochemical characteristics of ND and COE-S1:ND complexes.

Structure	Zeta potential (mV)	Hydrodynamic size (nm)	PDI
ND	-77.5	200.2	0.127
COE-S1D:ND	-48.2	433.6	0.25

Additionally, the COE-S1D:ND complexes showed a higher polydispersity index (PDI = 0.251) compared to the ND alone (PDI = 0.127), indicating a wider variation in particle sizes after complex formation (**Table 1**). These observations suggest partial aggregation and/or heterogeneous adsorption of protein molecules on the ND surface. Nevertheless, the final PDI remained below 0.3, indicating that the COE-S1D:ND complexes maintained an acceptable level of colloidal stability for vaccine applications.

The morphology and structural features of COE-S1D:ND complexes were assessed by TEM (**Figure 3E**). TEM images revealed that COE-S1D:ND had irregular morphologies. The observed heterogeneity in particle size and shape may stem from differences in protein adsorption levels or partial aggregation occurring during complex formation. Furthermore, TEM observations imply that single COE-S1D protein molecules may bridge two distinct nanoparticles, leading to the clustered appearance observed in the micrographs. Collectively, these results demonstrate that the COE-S1D-pII protein was effectively attached to the ND surfaces, resulting in the formation of COE-S1D:ND complexes as intended.

### The COE-S1:ND mixture induced stronger humoral and mucosal responses against PEDV than the free COE-S1D-pII protein in pregnant sows

**Figure 4:**
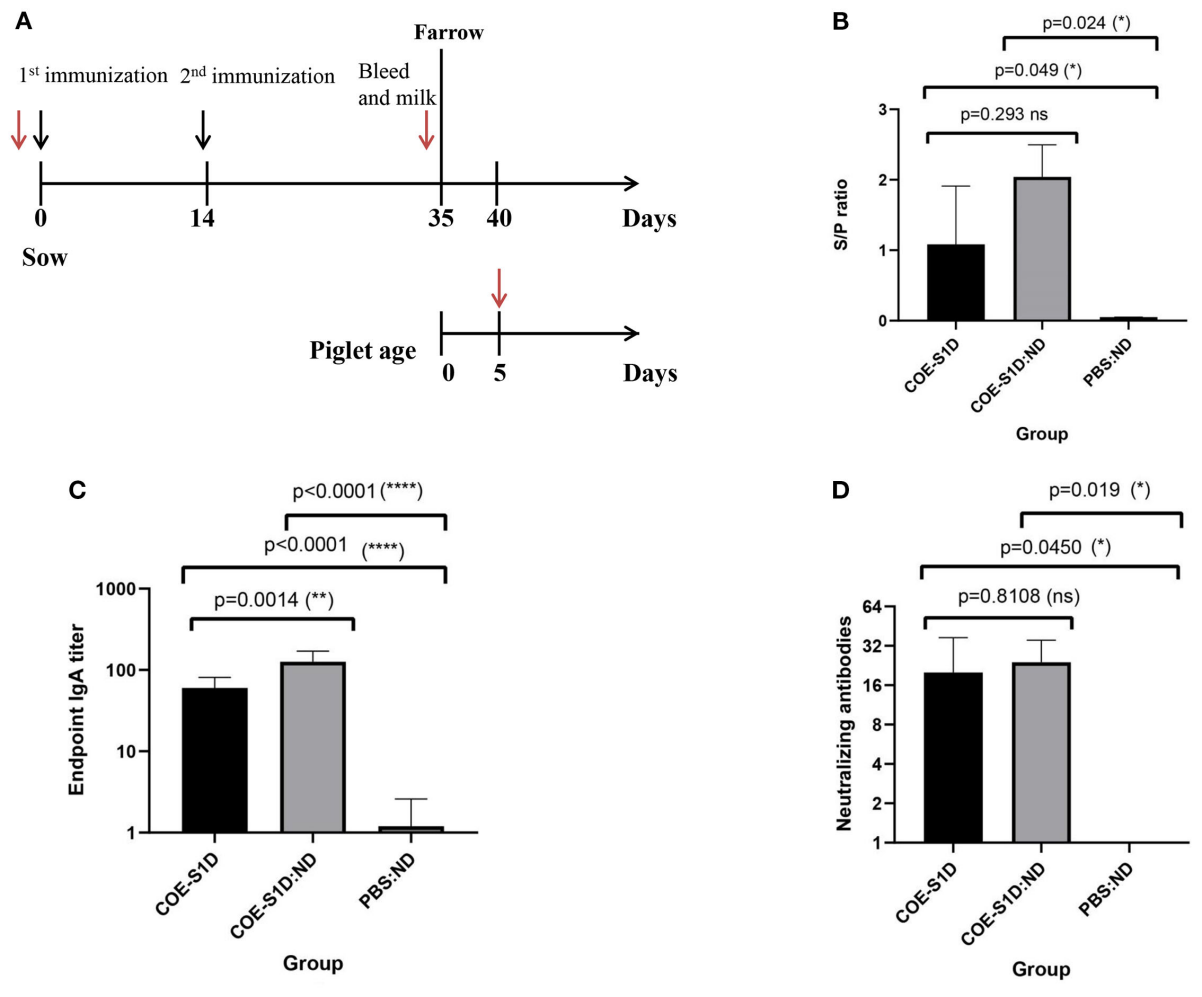
Humoral and mucosal immune responses in pregnant sows vaccinated with COE-S1D-pII protein or COE-S1D:ND complexes or PBS:ND. **(A)** Diagram illustrating the immunization schedule for pregnant sows (approximately day 80 of gestation, n=2 per group) and their piglets (n=5 per group). Black arrows indicate vaccination time points, while red arrows show blood sampling times. **(B)** PEDV-specific IgG concentrations in sera from sows immunized with COE-S1D-pII, COE-S1D:ND complexes, or PBS: ND controls were evaluated using a commercial ELISA kit. Samples with S/P ratios above 0.35 were considered positive for PEDV-specific IgG. **(C)** COE-specific IgA antibody levels in sow milk were determined by ELISA, using SEC-purified COE-S1D-pII protein as the capture antigen. **(D)** Neutralizing antibody titers in sow sera were evaluated using a virus-neutralization assay with the highly virulent PEDV G2b strain (100 TCID50/0.1 mL). VN titers ≥ 8 were considered indicative of PEDV-neutralizing antibodies. Data are shown as mean ± standard deviation (SD). Statistical significance was defined as p < 0.05. Levels of statistical significance are indicated as follows: *p < 0.05; **p < 0.01; **** p < 0.0001. ns, not significant.

The immunogenicity of the COE-S1D-pII protein and the mixture of COE-S1D protein and ND (1:24, w/w) were assessed in pregnant sows according to the immunization protocol shown in **Figure 4A**. On day 35 post-immunization (pi), PEDV-specific IgG antibodies were detected in the serum of the sow immunized with COE-S1D-pII, with an S/P mean ratio of 1.03 (**Figure 4B**). Meanwhile, the sow vaccinated with COE-S1D:ND showed a higher PEDV-specific IgG response, with a mean S/P ratio of 2.04. However, the difference in PEDV-specific IgG antibodies between the two groups was not statistically significant (p = 0.293 > 0.05). In contrast, no PEDV-specific IgG antibodies were detected in the serum of the control sow that received PBS: ND.

Similarly, on day 35 , pi, COE-S1D-specific IgA antibodies were detected in the milk of the sows vaccinated with COE-S1D-pII, with a mean endpoint IgA titer of 60 (**Figure 4C**). In comparison, the sows immunized with COE-S1D:ND exhibited a 2-fold higher IgA response, reaching a mean endpoint IgA titer of 126. This difference in COE-S1D-specific IgA antibodies between the two groups was statistically significant (p = 0.0014 < 0.01). In contrast, no COE-S1D-specific IgA antibodies were detected in the serum of the PBS: ND control sows.

Neutralizing antibody titers against a highly virulent PEDV G2b strain were measured using a virus neutralization assay. On day 35 post-immunization (pi), the sows immunized with COE-S1D-pII developed a virus-neutralizing mean titer of 20 (**Figure 4D**), whereas the sows vaccinated with COE-S1D:ND showed a slightly higher VN mean titer of 24. However, the difference in neutralizing antibody titers between the two groups was not statistically significant (p = 0.8108 > 0.05). In contrast, no neutralizing antibodies against PEDV were detected in the serum of the PBS: ND control sows.

Overall, these findings indicate that vaccination with COE-S1D:ND induced more robust humoral and muscosal immune responses in pregnant sows compared to immunization with COE-S1D-pII protein alone. In particular, a statistically significant increase in IgA levels was observed in the COE-S1D:ND group. Although IgG and neutralizing antibody titers tended to be higher in the COE-S1D:ND group, the differences were not statistically significant.

### COE-S1D:ND complex outperforms COE-S1D protein in inducing humoral and mucosal immunity in piglets

**Figure 5:**
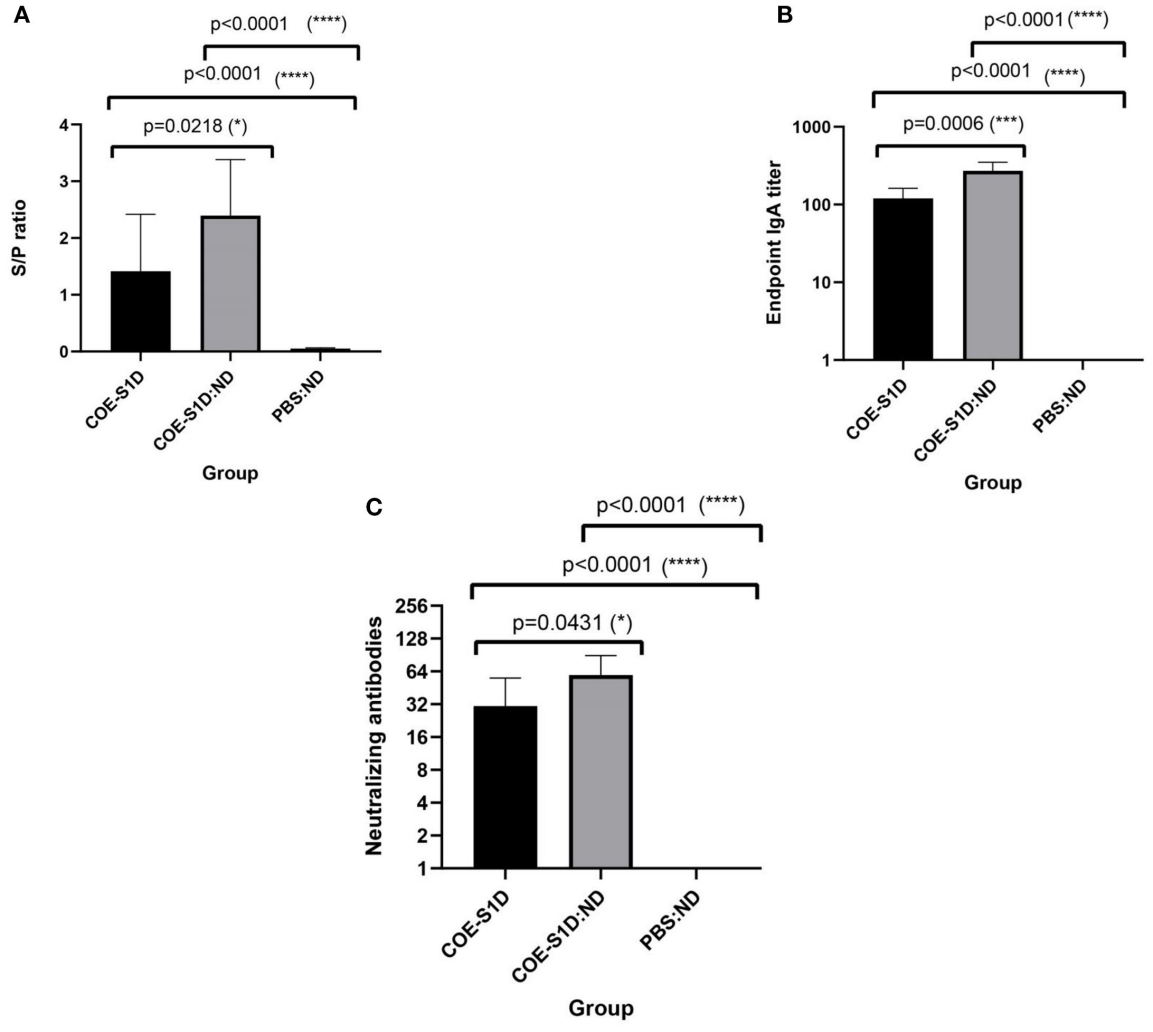
Evaluation of maternally derived antibodies in piglets born to immunized sows at day 5 postpartum. Data are expressed as mean ± standard deviation (SD). A statistically significant difference is defined as one where p < 0.05. **(A)** Levels of PEDV-specific IgG in sera of piglets born to sows (n=5 per group) immunized with COE-S1D-pII or COE-S1D:ND or PBS, quantified using a commercial ELISA kit. Samples with S/P ratios greater than 0.35 were classified as positive for PEDV-specific IgG. **(B)** COE-S1D-specific IgA concentrations in piglet sera (n = 5 per group) were determined by ELISA, using SEC-purified COE-S1D-pII protein as the coating antigen. **(C)** Virus-neutralizing antibody titers in piglet sera were measured via a virus neutralization assay against the highly virulent PEDV G2b strain (100 TCID50/0.1 ml). A VN titer ≥ 8 was considered indicative of the presence of PEDV-neutralizing antibodies. Levels of statistical significance are indicated as follows: *p < 0.05; ***p < 0.001; ****p < 0.0001.

As antibodies present in colostrum and milk from vaccinated sows can be transferred to piglets through suckling, passive immunity in the offspring was evaluated using ELISA and virus-neutralization assays. PEDV-specific IgG antibodies were detected in the sera of 5-day-old piglets born to the sows immunized with COE-S1D-pII. PEDV-specific IgG antibodies were detected in the serum of piglets born to the sows immunized with COE-S1D-pII, with an S/P mean ratio of 1.41. Meanwhile, the piglets born to the sows vaccinated with COE-S1D:ND showed a higher PEDV-specific IgG response, reaching an S/P mean ratio of 2.39. The difference in PEDV-specific IgG antibodies between the two groups was statistically significant (p = 0.0218 < 0.05). In contrast, no PEDV-specific IgG antibodies were detected in the serum of the control piglets born to the sows that received PBS: ND (**Figure 5A**).

Similarly, COE-S1D-specific IgA antibodies were present at high levels in the sera of these 5-day-old piglets born to the sows vaccinated with COE-S1D-pII, with a mean endpoint IgA titer of 120. In comparison, the sows immunized with COE-S1D:ND exhibited a 2.3-fold higher IgA response, reaching a mean endpoint IgA titer of 272. The difference in COE-S1D-specific IgA antibodies between the two groups was statistically significant (p = 0.0006 < 0.001). In contrast, no COE-S1D-specific IgA antibodies were detected in the serum of the PBS: ND control piglet (**Figure 5B**).

Virus-neutralizing antibodies against a highly virulent PEDV G2b strain were also identified in sera of these 5-day-old piglets. The piglets born to the sows immunized with COE-S1D-pII had a virus-neutralizing titer of 30.8, whereas the piglets born to the sows vaccinated with COE-S1D:ND showed a 1.6-fold higher VN mean titer of 49.6. The difference in virus-neutralizing antibodies between the two groups was statistically significant (p = 0.0431 < 0.05). In contrast, no neutralizing antibodies against PEDV were detected in the serum of the PBS: ND control piglets (**Figure 5C**).

Taken together, these results demonstrate successful passive transfer of PEDV-specific IgG, COE-specific IgA, and neutralizing antibodies from the immunized pregnant sows to their piglets via colostrum and milk. Furthermore, piglets born to sows immunized with the COE-S1D:ND complex consistently exhibited stronger antibody responses compared with piglets from sows immunized with COE-S1D-pII protein.

## Discussion

NDs belong to a class of carbon-based nanomaterials characterized by a sp³ crystalline lattice, the same structure that imparts natural diamonds with remarkable hardness and insulating properties. In addition to their physical characteristics, nanodiamonds (NDs) are highly regarded in biomedical research for their excellent biocompatibility, stability *in vivo*, and flexible surface chemistry that can be tailored for a wide range of applications (37). Within vaccinology, NDs present several unique benefits. Their nanoscale size is comparable to that of many bacterial and viral pathogens, enabling them to serve as both adjuvants that boost immune responses and as carriers that enhance antigen presentation (38, 39). Furthermore, the hydrophobic properties of acid-oxidized NDs facilitate strong interactions with native membrane proteins as well as soluble antigens (40).

Plant-based expression systems have been widely used to produce a variety of recombinant proteins, vaccines, and bioactive compounds (17, 41, 42). Plant expression platforms using transient offer several advantages, including rapid protein accumulation, relatively consistent yields, scalability, and cost-effectiveness compared with microbial or mammalian systems (17, 41–44). However, some limitations remain. Protein expression can be variable and occasionally lower than in chemical or microbial systems (45), and downstream purification is often complicated by plant-derived compounds such as phenolics, lignin, and polysaccharides, which can interfere with chromatography and reduce product purity (46). Moreover, plant-specific glycosylation patterns may differ from those of humans and animals, potentially affecting protein activity or immunogenicity (47). Despite these challenges, transient plant-based expression remains a versatile and valuable platform, as illustrated by the successful development of plant-derived vaccines. The Newcastle disease vaccine for poultry, produced in plants, was the first to receive approval by the U.S. Department of Agriculture in 2006. Since then, vaccines against influenza, Ebola, rabies, hepatitis B, norovirus, anthrax, and rotavirus have advanced through clinical trials. Medicago’s quadrivalent influenza vaccine completed Phase 3 trials, demonstrating safety, efficacy, and strong immunogenicity in humans (48). Building on the strengths and limitations of plant-based expression platforms as well as our previous experience in expressing various recombinant proteins in plants such as hemagglutinin protein from H5N1 (49, 50), H7N9 (28), and the COE protein from PEDV (19, 20), we for the first time produced COE-S1D-pII protein in *N. benthamiana* via transient expression and investigated the effect of nanodiamonds on the immune response to this plant-derived protein in pigs. Here, we utilized acid-oxidized-ND as carriers to present the COE-S1D protein produced in plants from PEDV. The successful coating of COE protein onto NDs was verified by Western blot analysis across a range of protein-to-nanoparticle mass ratios. The highest adsorption efficiency was observed at a ratio of 1:24 (w/w), as confirmed by Western blot signals. Analysis by dynamic light scattering revealed a significant enlargement in particle size and a change in zeta potential in the COE-S1D:ND complexes, compared with bare ND nanoparticles. These results confirm that the COE-S1D protein can effectively coat the surface of ND without the need for chemical crosslinking agents. Comparable adsorption behavior has been documented, such as the binding of OVA to solid NDs without the need for additional coupling reagents (28, 51, 52). In earlier work, the binding of glycoprotein H7-pII protein to ND caused only a slight shift in zeta potential (from approximately –45 mV to –38 mV), and the hydrophobic regions of the H7-pII protein were thought to mediate adsorption, thereby preserving glycosylated epitopes crucial for immune recognition (28). Considering these structural similarities, it is plausible that COE-S1D attaches to ND nanoparticles mainly via hydrophobic interactions, rather than through electrostatic attraction or direct binding to glycans. This type of adsorption is likely to preserve the native antigenic structure of COE-S1D, potentially improving its recognition by immune cells. Thus, non-covalent coating onto ND represents an effective strategy for formulating COE-S1D:ND vaccine complexes without chemical modification.

The immunogenicity of the COE-S1D:ND mixture was assessed in pregnant sows in comparison with the free COE-S1D protein. Vaccination with the COE-S1D:ND complexes elicited a stronger humoral response against PEDV than the free COE-S1D-pII formulation in pregnant sows. Intramuscular immunization of pregnant sows with the COE-S1D-pII protein and the COE-S1D:ND complexes primarily induced systemic humoral responses, as evidenced by elevated PEDV-specific IgG levels. Specifically, the sows receiving the COE-S1D:ND mixture developed a PEDV-specific IgG response with an S/P mean ratio approximately twice that observed in the free COE-S1D-pII group. Importantly, we also detected increased IgA titers in sow milk. Since the porcine placenta does not transfer antibodies during gestation, newborn piglets acquire maternal antibodies almost exclusively by ingesting colostrum and milk after birth (53). Higher concentrations of secretory IgA in maternal milk play a crucial role in protecting piglets against enteric pathogens, including PEDV (8, 54, 55). Notably, a twofold higher endpoint IgA titer was also detected in the milk of the sows vaccinated with the COE-S1D:ND mixture compared to sows immunized with the COE-S1D protein alone. These findings are in line with previous reports indicating that intramuscular immunization has been shown to elicit antigen-specific IgG responses while also promoting IgA production (56). In addition, multiple studies highlight the essential role of both IgA and IgG antibodies in safeguarding piglets against PEDV infection (57, 58). In addition, the sows vaccinated with the COE-S1D:ND mixture showed a modestly higher virus-neutralizing antibody titer relative to those immunized with the free COE-S1D-pII protein. Considering the well-established correlation between neutralizing antibody titers and vaccine efficacy documented in commercial vaccines (59), improving neutralizing antibody responses after vaccination remains an essential goal in vaccine design and development.

Next, the humoral immune response against PEDV was observed in piglets born to sows vaccinated with either the COE-S1D-pII protein or the COE-S1D:ND complexes. Notably, higher PEDV-specific IgG antibodies were detected in piglets from sows vaccinated with the COE-S1D:ND complexes compared to those from sows vaccinated with the free COE-S1D-pII protein. In addition, piglets in the COE-S1D:ND group showed a twofold higher endpoint titer of COE-S1D-specific IgA antibodies and a 1.6-fold higher level of PEDV-neutralizing antibodies. The presence of IgA in piglet serum further suggests effective transfer of these antibodies, contributing to systemic and mucosal immunity (60). Overall, these results suggest that the COE-S1D:ND formulation may enhance both humoral and mucosal immune responses, which are crucial for protecting newborn piglets against PEDV infection.

Interestingly, the humoral immune responses against PEDV measured in 5-day-old piglets were higher than those observed in the mother sows. Before parturition, sows actively transport large amounts of IgG and IgA from the bloodstream into the mammary glands, leading to colostrum antibody concentrations several times higher than those in maternal serum. This selective transfer temporarily lowers antibody levels in the sow’s blood after farrowing. As a result, the serum concentrations of IgG, IgA, and virus-neutralizing antibodies in piglets can temporarily exceed those in the sow’s serum and milk at the same time point, especially within the first few days postpartum. Five-day-old piglets retain high levels of antibodies absorbed earlier from colostrum, so their serum IgG, IgA, and neutralizing antibody levels can remain higher than those measured in the sow’s serum and milk sampled concurrently. Our observations agree with prior research indicating that IgG and IgA concentrations in piglets generally follow similar trends to those in sow serum and colostrum (53). Although the serum antibody levels in 5-day-old piglets can temporarily exceed those measured in the sows immediately after farrowing, the overall humoral responses in piglets still reflect the antibody status of their mothers.

Several studies found that high levels of neutralizing antibodies play a crucial role in safeguarding against PEDV infections (21, 61). In the present study, we aimed to develop an improved formulation capable of eliciting stronger immune responses, particularly neutralizing activity against PEDV G2b, which is the predominant circulating strain in Vietnam. When comparing the neutralizing antibody titers against PEDV, piglets born to sows vaccinated with the free COE-S1D protein showed approximately 3.8-fold higher titers than the titer reported in piglets born to sows vaccinated with the COE-pII protein in the previous study (20), in which the COE-pII protein elicited protective immunity in piglets against PEDV G2a. Furthermore, when comparing the immunogenicity of COE-pII protein and COE-S1D-pII protein in piglets, we observed that piglets immunized with COE-S1D-pII protein produced stronger COE-specific IgG, IgA, and neutralizing antibody responses against PEDV G2a than those vaccinated with COE-pII protein (data not shown). These results indicate that COE-S1D-pII could represent a stronger correlate of protection, underscoring its promise for developing more effective vaccines against PED in Vietnam. In another experiment, piglets immunized with 50 µg of COE-S1D-pII protein developed COE-S1D-specific IgG and IgA, as well as neutralizing antibodies against PEDV, comparable to those induced by a commercial PEDV vaccine after two immunizations (**Supplementary Figure 1**). In addition, piglets born to sows vaccinated with the COE-S1D:ND mixture showed approximately 6.2-fold higher titers than the titers in piglets born to sows vaccinated with the COE-pII protein in the previous study (20). The neutralizing antibody titer found in piglets vaccinated with the COE-S1D mixture was quite similar to that found in piglets vaccinated with COE-ferritin, one of the ferritin nanoparticle-based vaccines in a recent study (62). As a result, incorporating ND nanoparticles markedly boosted the immunogenicity of the plant-produced COE-S1D protein. The new formulation markedly enhanced both systemic and mucosal immunity and significantly increased neutralizing antibody titers in piglets. The current formulation may provide more effective coverage against prevalent PEDV G2b strain.

Various studies have demonstrated that antigens can be adsorbed onto ND nanoparticles can markedly increase humoral immune responses and immunological benefits (28, 51, 63, 64). Nanocomplexes are formed by conjugating viral proteins such as HA/H7N9 onto oxidized NDs with particle sizes from roughly 50 nm to 500 nm. These complexes significantly boosted vaccine immunogenicity: hemagglutination titers increased up to 512-fold compared to the free H7 protein, while H7-specific IgG levels in mice rose by over 15.4-fold after the second immunization (28). Similarly, negatively oxidized NDs conjugated with HA/H5N1 antigen led to higher hemagglutination titers and elevated HA-specific IgG and neutralizing antibodies in mice (51). Beyond viral proteins, NDs have also been combined with other bioactive molecules to enhance immune responses further. For instance, carboxylated NDs covalently linked to NH2-PLGA nanoparticles encapsulating fig polysaccharides (FP) formed NDs-PLGA-FP/OVA complexes. These complexes promoted antigen uptake, increased lymphocyte proliferation, elevated expression of MHC II, CD80, and CD86, and shifted the Th1/Th2 cell balance. They also activated the IL-17 signaling pathway, resulting in higher secretion of cytokines and leading to increased OVA-specific IgG levels (65). Importantly, NDs and fluorescent NDs (FNDs) have demonstrated safety and efficacy as nonallergenic adjuvants when combined with incomplete Freund’s adjuvant (IFA). In a mouse model immunized with ovalbumin (OVA), these ND-based formulations significantly enhanced antibody production and inhibited tumor growth, maintaining suppression of lymphoma cells for over 35 days (52). The exact ways nanodiamonds (NDs) affect antigen presentation and stimulate immune cells remain unclear. While our study primarily evaluated immunogenicity, previous research provides indirect evidence for the immunostimulatory potential of NDs. NDs and nanoplatinum have been shown to activate dendritic cells *in vitro*, promoting CD4+ T cell proliferation and up-regulation of CD25, indicative of adaptive immune activation (66). NDs also were demonstrated to trigger receptors on dendritic cells and macrophages, inducing strong antiviral-like responses while preserving cell viability (67). These observations suggest pathways for NDs to enhance antigen presentation and immune activation, yet further mechanistic work is needed. Taken together, these studies highlight the potential of NDs as versatile and biocompatible platforms for improving vaccine efficacy.

Even though our data showed that the COE-S1D:ND formulation improved immunogenicity, there are a few limitations worth noting. It is important to note that PEDV causes the highest mortality in piglets younger than one week of age (3). While our main focus was on the immune responses of piglets born to sows immunized with the COE-S1D-pII protein or the COE-S1D:ND complexes, the limited number of sows constrained the statistical power of our analyses. In our study, each sow produced five piglets, and the immune response data from these piglets provided sufficient statistical power for meaningful analysis. Moreover, the use of a limited number of sows in immunization trials was reported in similar studies (2, 36), supporting the feasibility of our experimental design. The main constraints contributing to this limitation were the high costs and logistical challenges associated with maintaining and immunizing pregnant sows and ethical considerations in using large animals. Additionally, at the time of the study, the widespread outbreak of African swine fever across many regions of Vietnam made it particularly difficult to recruit herds that were both healthy and PEDV-negative. Moreover, due to the high cost of long-term monitoring, we could not extend the follow-up period in this study, which is important to determine the duration of protection conferred by the vaccine. Here, we did not assess cellular immune responses, such as T cell activation. Nevertheless, our previous research showed that another plant-based COE-pII protein could induce IFN-γ responses in both sows and their piglets (20). Additionally, challenge experiments were not conducted to directly evaluate protective efficacy, including survival or mortality after exposure to highly virulent PEDV strains. Nevertheless, the neutralizing antibody titers observed in piglets from sows immunized with the COE-S1D-pII protein and the COE-S1D:ND complexes were higher than those previously shown to confer piglet protection, suggesting promising vaccine potential. Future studies should involve larger cohorts of sows and incorporate assessments of long-term immunity, cellular immune responses, and challenge experiments to evaluate this nanovaccine candidate’s protective potential fully. Moreover, to enable large-scale production of COE-S1D-pII protein, further optimization of the purification process is necessary, using methods suitable for scalable manufacturing. These efforts aim to establish a nanovaccine candidate capable of inducing broader and longer-lasting immunity against PEDV strains circulating in Vietnam.

## Conclusion

In summary, the plant-expressed COE-S1D-pII protein was efficiently bound to the surface of ND nanoparticles. The resulting COE-S1D:ND formulation (1:24, w/w) triggered markedly stronger humoral immune responses than the free COE-S1D-pII protein, including elevated PEDV-specific IgG and IgA levels, as well as higher neutralizing antibody titers against a highly virulent PEDV G2b strain in piglets. These findings indicate that nanodiamonds can enhance the immunogenicity of the plant-derived COE-S1D-pII protein. Overall, this highlights the potential of integrating plant-based subunit antigens with nanotechnology to support the development of more effective vaccines against PEDV.

## Data Availability

The original contributions presented in the study are included in the article/**Supplementary Material**. Further inquiries can be directed to the corresponding authors.

## Supplementary material

Supplementary Figure 1 Humoral immune responses in piglets induced by COE-S1D-pII protein and a commercial vaccine. **(A)**. Piglet immunization scheme experiment. COE-S1D-pII protein (50 µg/dose) mixed Emulsigen^®^-D adjuvant (MVP) or inactivated vaccine containing the PEDV AJ1102 + TGEV WH-1 strains (Keqian Biology, Wuhan Keqian Biology, China) or PBS mixed Emulsigen^®^-D adjuvant (MVP) was immunized in piglets. The red arrow and blue arrow show bleeding time and vaccination, respectively. **(B)**. COE-S1D-specific IgG antibodies in piglet sera were evaluated by ELISA using SEC-purified COE-S1D-pII as antigen. Data are presented as mean ± Standard deviation (SD). *indicates a statistically significant difference (p< 0.05). **(C)**. COE-S1D-specific IgA antibodies in piglet sera were evaluated by ELISA using SEC-purified COE-S1D-pII as antigen. Data are presented as mean ± Standard deviation (SD). *indicates a statistically significant difference (p< 0.05). **(D)**. Neutralizing antibodies in piglet sera were analyzed by the virus-neutralizing assay. PEDV SM98 (10^3^ TCID50/0.1 mL) was used for the assay. A VN titer equal to or greater than 8 was considered indicative of a positive presence of neutralizing antibodies against PEDV.

## References

[1] Song D Park B. Porcine epidemic diarrhoea virus: a comprehensive review of molecular epidemiology, diagnosis, and vaccines. Virus Genes. (2012) 44:167–75. doi: 10.1007/s11262-012-0713-1, PMID: 22270324 PMC7089188

[2] Zhao Y Fan B Song X Gao J Guo R Yi C. PEDV-spike-protein-expressing mRNA vaccine protects piglets against PEDV challenge. mBio. (2024) 15:e02958. doi: 10.1128/mbio.02958-23, PMID: 38231557 PMC10865985

[3] Jung K Saif LJ Wang Q. Porcine epidemic diarrhea virus (PEDV): an update on etiology, transmission, pathogenesis, and prevention and control. Virus Res. (2020) 286:198045. doi: 10.1016/j.virusres.2020.198045, PMID: 32502552 PMC7266596

[4] Li W Li H Liu Y Pan Y Deng F Song Y. New variants of porcine epidemic diarrhea virus, China, 2011. Emerg Infect Dis. (2012) 18:1350–3. doi: 10.3201/eid1808.120002, PMID: 22840964 PMC3414035

[5] Song D Moon H Kang B. Porcine epidemic diarrhea: a review of current epidemiology and available vaccines. Clin Exp Vaccine Res. (2015) 4:166–76. doi: 10.7774/cevr.2015.4.2.166, PMID: 26273575 PMC4524901

[6] Zhang Y Chen Y Yuan W Peng Q Zhang F Ye Y. Evaluation of cross-protection between G1a- and G2a-genotype porcine epidemic diarrhea viruses in suckling piglets. Anim (Basel). (2020) 10:1674. doi: 10.3390/ani10091674, PMID: 32957461 PMC7552732

[7] Than VT Choe SE Vu TTH Do TD Nguyen TL Bui TTN. Genetic characterization of the spike gene of porcine epidemic diarrhea viruses (PEDVs) circulating in Vietnam from 2015 to 2016. Vet Med Sci. (2020) 6:535. doi: 10.1002/vms3.256, PMID: 32159913 PMC7397879

[8] Langel SN Paim FC Lager KM Vlasova AN Saif LJ. Lactogenic immunity and vaccines for porcine epidemic diarrhea virus (PEDV): historical and current concepts. Virus Res. (2016) 226:93–107. doi: 10.1016/j.virusres.2016.05.018, PMID: 27212686 PMC7111331

[9] Liu J Shi H Chen J Zhang X Ji Z Yuan J. Neutralization of genotype 2 porcine epidemic diarrhea virus strains by a novel monoclonal antibody. Virology. (2017) 507:257–62. doi: 10.1016/j.virol.2017.04.017, PMID: 28463713 PMC7172788

[10] Sun D Feng L Shi H Chen J Liu S Chen H. Spike protein region (aa 636–789) of porcine epidemic diarrhea virus is essential for induction of neutralizing antibodies. Acta Virol. (2007) 51:149–56., PMID: 18076304

[11] Sun J Li Q Shao C Ma Y He H Jiang S. Isolation and characterization of Chinese porcine epidemic diarrhea virus with novel mutations and deletions in the S gene. Vet Microbiol. (2018) 221:81–9. doi: 10.1016/j.vetmic.2018.05.021, PMID: 29981713 PMC7117340

[12] Hsueh FC Lin CN Chiou HY Chia MY Chiou MT Haga T. Updated phylogenetic analysis of the spike gene and identification of a novel recombinant porcine epidemic diarrhoea virus strain in Taiwan. Transbound Emerg Dis. (2020) 67:417–30. doi: 10.1111/tbed.13365, PMID: 31538715

[13] Piao DC Shin DW Kim IS Li HS Oh SH Singh B. Trigger factor assisted soluble expression of recombinant spike protein of porcine epidemic diarrhea virus in Escherichia coli. BMC Biotechnol. (2016) 16:39. doi: 10.1186/s12896-016-0269-y, PMID: 27142206 PMC4855837

[14] Park SM Mo AY Lim JG Chung HJ Kim TG Kim KJ. Surface displayed expression of a neutralizing epitope of spike protein from a Korean strain of porcine epidemic diarrhea virus. Biotechnol Bioprocess Eng. (2007) 12:690–5. doi: 10.1007/BF02931087, PMID: 32218674 PMC7090475

[15] Do VT Jang J Park J Dao HT Kim K Hahn TW. Recombinant adenovirus carrying a core neutralizing epitope of porcine epidemic diarrhea virus and heat-labile enterotoxin B of Escherichia coli as a mucosal vaccine. Arch Virol. (2020) 165:609–18. doi: 10.1007/s00705-019-04474-y, PMID: 31950289 PMC7087028

[16] Huy NX Tien NQ Kim MY Kim TG Jang YS Yang MS. Immunogenicity of an S1D epitope from porcine epidemic diarrhea virus and cholera toxin B subunit fusion protein transiently expressed in infiltrated *Nicotiana benthamiana* leaves. Plant Cell Tissue Organ Cult. (2016) 127:369–80. doi: 10.1007/s11240-016-1059-5, PMID: 32214565 PMC7088629

[17] Rybicki EP. Plant-made vaccines for humans and animals. Plant Biotechnol J. (2010) 8:620–37. doi: 10.1111/j.1467-7652.2010.00507.x, PMID: 20233333 PMC7167690

[18] Topp E Irwin R McAllister T Lessard M Joensuu JJ Kolotilin I. The case for plantmade veterinary immunotherapeutics. Biotechnol Adv. (2016) 34:597–604. doi: 10.1016/j.bioteChadv.2016.02.007, PMID: 26875776

[19] Ho TT Nguyen GT Pham NB Le VP Trinh TBN Vu TH. Plant-derived trimeric CO-26K-equivalent epitope induced neutralizing antibodies against porcine epidemic diarrhea virus. Front Immunol. (2020) 11:2152. doi: 10.3389/fimmu.2020.02152, PMID: 33042128 PMC7524870

[20] Ho TT Trinh VT Tran HX Le PTT Nguyen TT Hoang HTT. The immunogenicity of plant-based COE-GCN4pII protein in pigs against the highly virulent porcine epidemic diarrhea virus strain from genotype 2. Front Vet Sci. (2022) 9:940395. doi: 10.3389/fvets.2022.940395, PMID: 35967993 PMC9366249

[21] Brown J Poonsuk K Cheng T-Y Rademacher C Kalkwarf E Tian L. Comparison of two diagnostic assays for the detection of serum neutralizing antibody to porcine epidemic diarrhea virus. Animals. (2023) 13:757. doi: 10.3390/ani13040757, PMID: 36830544 PMC9951927

[22] Asbach B Wagner R. Particle-based delivery of the HIV envelope protein. Curr Opin HIV AIDS. (2017) 12:265. doi: 10.1097/COH.0000000000000366, PMID: 28422790

[23] Anselmo AC Mitragotri S. Nanoparticles in the clinic: An update. Bioeng Transl Med. (2019) 4:e10143. doi: 10.1002/btm2.10143, PMID: 31572799 PMC6764803

[24] Zhang X Hu W Li J Tao L Wei Y. A comparative study of cellular uptake and cytotoxicity of multi-walled carbon nanotubes, graphene oxide, and nanodiamond. Toxicol Res. (2012) 1:62–8. doi: 10.1039/c2tx20006f

[25] Mochalin VN Shenderova O Ho D Gogotsi Y. The properties and applications of nanodiamonds. Nat Nanotechnol. (2012) 7:11–23. doi: 10.1038/nnano.2011.209, PMID: 22179567

[26] Wang L Su W Ahmad KZ Wang X Zhang T Yu Y. Safety evaluation of nanodiamond-doxorubicin complexes in a Naïve Beagle canine model using hematologic, histological, and urine analysis. Nano Res. (2022) 15:3356–66. doi: 10.1007/s12274-021-3867-0

[27] Alexander E Leong KW. Toxicity and biodistribution comparison of functionalized nanodiamonds, quantum dot nanocarbons and gold nanoparticles. Front Nanotechnol. (2025) 7:1512622. doi: 10.3389/fnano.2025.1512622

[28] Pham NB Ho TT Nguyen GT Le TT Le NT Chang HC. Nanodiamond enhances immune responses in mice against recombinant HA/H7N9 protein. J Nanobiotechnol. (2017) 15:69. doi: 10.1186/s12951-017-0318-2, PMID: 28982373 PMC5629800

[29] Seaberg J Clegg JR Bhattacharya R Mukherjee P. Self-therapeutic nanomaterials: applications in biology and medicine. Mater Today. (2023) 62:190–224. doi: 10.1016/j.mattod.2022.11.007, PMID: 36938366 PMC10022599

[30] Pham VD Hoang H Phan HT Conrad U Chu HH. Production of antibody labeled gold nanoparticles for influenza virus H5N1 diagnosis kit development. Adv Nat Sci Nanosci Nanotechnol. (2012) 3:45017. doi: 10.1088/2043-6262/3/4/045017

[31] Weldon WC Wang BZ Martin MP Koutsonanos DG Skountzou I. Enhanced immunogenicity of stabilized trimeric soluble influenza hemagglutinin. PloS One. (2010) 5:e12466. doi: 10.1371/journal.pone.0012466, PMID: 20824188 PMC2931692

[32] Waterhouse A Bertoni M Bienert S Studer G Tauriello G Gumienny R. SWISS-MODEL: homology modelling of protein structures and complexes. Nucleic Acids Res. (2018) 46:W296–W303. doi: 10.1093/nar/gky427, PMID: 29788355 PMC6030848

[33] Ho TT Tran HT Nguyen LD Nghiem LHT Nguyen HD Nguyen HTT. The effect of silica nanoparticles on the immunogenicity of plant-based recombinant COE protein of porcine epidemic diarrhea virus in mice. J Nanopart Res. (2025) 27:157. doi: 10.1007/s11051-025-06351-w

[34] Wrapp D McLellan JS. The 3.1-Angstrom cryo-electron microscopy structure of the porcine epidemic diarrhea virus spike protein in the prefusion conformation. J Virol. (2019) 93:e00923-19. doi: 10.1128/jvi.00923-19, PMID: 31534041 PMC6854500

[35] Huang CY Draczkowski P Wang YS Chang CY Chien YC Cheng YH. *In situ* structure and dynamics of an alphacoronavirus spike protein by cryo-ET and cryo-EM. Nat Commun. (2022) 13:4877. doi: 10.1038/s41467-022-32588-3, PMID: 35986008 PMC9388967

[36] Makadiya N Brownlie R van den Hurk J Berube N Allan B Gerdts V. S1 domain of the porcine epidemic diarrhea virus spike protein as a vaccine antigen. Virol J. (2016) 13:57. doi: 10.1186/s12985-016-0512-8, PMID: 27036203 PMC4818391

[37] Alexander E Leong KW. Nanodiamonds in biomedical research: therapeutic applications and beyond. PNAS Nexus. (2024) 3:198. doi: 10.1093/pnasnexus/pgae198, PMID: 38983694 PMC11231952

[38] Xiang SD Scholzen A Minigo G David C Apostolopoulos V. Pathogen recognition and development of particulate vaccines: does size matter? Methods. (2006) 40:1–9. doi: 10.1016/j.ymeth.2006.07.018, PMID: 16997708

[39] Zhao L Seth A Wibowo N Zhao CX Mitter N. Nanoparticle vaccines. Vaccine. (2014) 32:327–37. doi: 10.1016/j.vaccine.2013.11.069, PMID: 24295808

[40] Pham MD Yu SSF Han CC Chan SI. Improved mass spectrometric analysis of membrane proteins based on rapid and versatile sample preparation on nanodiamond particles. Anal Chem. (2013) 85:6748–55. doi: 10.1021/ac4012207, PMID: 23763332

[41] Burnett MJ Burnett AC. Therapeutic recombinant protein production in plants: Challenges and opportunities. Plants People Planet. (2020) 2:121–32. doi: 10.1002/ppp3.10073

[42] Kulshreshtha A Sharma S Padilla CS Mandadi KK. Plant-based expression platforms to produce high-value metabolites and proteins. Front Plant Sci. (2022) 13:1043478. doi: 10.3389/fpls.2022.1043478, PMID: 36426139 PMC9679013

[43] Sainsbury F Thuenemann EC Lomonossoff GP. Plant-based expression systems for recombinant protein production. Plant Biotechnol J. (2010) 8:588–93. doi: 10.1111/j.1467-7652.2010.00500.x, PMID: 20331532

[44] Gleba Y Klimyuk V Marillonnet S. Plant viral vectors for delivery by Agrobacterium. Curr Opin Plant Biol. (2014) 19:16–23. doi: 10.1016/j.pbi.2014.02.001, PMID: 24631844

[45] Taghizadeh MS Abbasi A Moghadam A Niazi A. Enhancing cyclotide bioproduction: harnessing biological synthesis methods and various expression systems for large-scale manufacturing. Transgenic Res. (2025) 45:836–58. doi: 10.1007/s11248-023-00341-1, PMID: 39510598

[46] Lico C Chen Q Spisni A. Plant expression systems: tools and applications. Biotechnol Adv. (2008) 26:381–403. doi: 10.1016/j.bioteChadv.2008.05.002, PMID: 18573633

[47] Gomord V Fitchette A-C Menu-Bouaouiche L Saint-Jore-Dupas C Plasson C Michaud D. Plant-specific glycosylation patterns in the context of therapeutic protein production. Plant Biotechnol J. (2005) 3:191–225. doi: 10.1111/j.1467-7652.2005.00103.x, PMID: 20233335

[48] Ward BJ Makarkov A Séguin A Pillet S Trépanier S Dhaliwall J. Efficacy, immunogenicity, and safety of a plant-derived, quadrivalent, virus-like particle influenza vaccine in adults (18-64 years) and older adults (≥65 years): two multicentre, randomised phase 3 trials. Lancet. (2020) 396:1491–1503. doi: 10.1016/S0140-6736(20)32014-6, PMID: 33065035

[49] Phan HT Ho TT Chu HH Vu TH Gresch U Conrad U. Neutralizing immune responses induced by oligomeric H5N1-hemagglutinins from plants. Vet Res. (2017) 48:53. doi: 10.1186/s13567-017-0458-x, PMID: 28931425 PMC5607582

[50] Phan HT Tran HX Ho TT Pham VT Trinh VT Nguyen TT. Plant crude extracts containing oligomeric hemagglutinins protect chickens against highly pathogenic avian influenza virus after one dose of immunization. Vet Res Commun. (2023) 47:191–205. doi: 10.1007/s11259-022-09942-3, PMID: 35633471 PMC9145123

[51] Ho TT Pham VT Nguyen TT Trinh VT Vi T Lin HH. Effects of size and surface properties of nanodiamonds on the immunogenicity of plant-based H5 protein of A/H5N1 virus in mice. Nanomaterials (Basel). (2021) 11:1597. doi: 10.3390/nano11061597, PMID: 34204514 PMC8234943

[52] Lin HH Wang CY Hsieh FJ Liao FZ Su YK Pham MD. Nanodiamonds-in-oil emulsions elicit potent immune responses for effective vaccination and therapeutics. Nanomedicine (Lond). (2023) 18:1045–59. doi: 10.2217/nnm-2023-0179, PMID: 37610004

[53] Bandrick M Ariza-Nieto C Baidoo SK Molitor TW. Colostral antibody-mediated and cell-mediated immunity contributes to innate and antigen-specific immunity in piglets. Dev Comp Immunol. (2014) 43:114–20. doi: 10.1016/j.dci.2013.11.005, PMID: 24252519 PMC3902642

[54] Song Q Stone S Drebes D Greiner LL Dvorak CMT Murtaugh MP. Characterization of anti-porcine epidemic diarrhea virus neutralizing activity in mammary secretions. Virus Res. (2016) 226:85–92. doi: 10.1016/j.virusres.2016.07.006, PMID: 27287711 PMC7126973

[55] Langel SN Paim FC Alhamo MA Buckley A Van Geelen A Lager KM. Stage of gestation at porcine epidemic diarrhea virus infection of pregnant swine impacts maternal immunity and lactogenic immune protection of neonatal suckling piglets. Front Immunol. (2019) 10:727. doi: 10.3389/fimmu.2019.00727, PMID: 31068924 PMC6491507

[56] Hsueh FC Chang YC Kao CF Hsu CW Chang HW. Intramuscular immunization with chemokine-adjuvanted inactive porcine epidemic diarrhea virus induces substantial protection in pigs. Vaccines (Basel). (2020) 8:102. doi: 10.3390/vaccines8010102, PMID: 32102459 PMC7157555

[57] Annamalai T Lin CM Gao X Liu X Lu Z Saif LJ. Cross-protective immune responses in nursing piglets infected with a US spike-insertion deletion porcine epidemic diarrhea virus strain and challenged with an original US PEDV strain. Vet Res. (2017) 48:61. doi: 10.1186/s13567-017-0469-7, PMID: 28985754 PMC6389210

[58] Krishna VD Kim Y Yang M Vannucci F Molitor T Torremorell M. Immune responses to porcine epidemic diarrhea virus (PEDV) in swine and protection against subsequent infection. PloS One. (2020) 15:e0231723. doi: 10.1371/journal.pone.0231723, PMID: 32343704 PMC7188253

[59] Zinkernagel RM. Maternal antibodies, childhood infections, and autoimmune diseases. N Engl J Med. (2001) 345:1331–5. doi: 10.1056/NEJMra012493, PMID: 11794153

[60] Hu Z Li Y Zhang B Zhao Y Guan R Zhou Y. Serum IgA antibody level against porcine epidemic diarrhea virus is a potential pre-evaluation indicator of immunization effects in sows during parturition under field conditions. Porc Health Manag. (2024) 10:32. doi: 10.1186/s40813-024-00382-w, PMID: 39228006 PMC11373460

[61] Hu G Luo X Liao J Zou C Huang Y Geng R. Neutralizing antibody levels as a key factor in determining the immunogenic efficacy of the novel PEDV alpha coronavirus vaccine. Vet Q. (2025) 45:1–20. doi: 10.1080/01652176.2025.2509506, PMID: 40432512 PMC12120861

[62] Liu Y Deng J Bi Z Luo M Han X Yao L. Ferritin-based nanoparticle vaccine protects neonatal piglets against porcine epidemic diarrhea virus challenge following immunization of pregnant sows. Vet Res. (2025) 56:140. doi: 10.1186/s13567-025-01542-8, PMID: 40624655 PMC12235928

[63] Kong XL Huang LCL Hsu CM Chen WH Han CC Chang HC. High-affinity capture of proteins by diamond nanoparticles for mass spectrometric analysis. Anal Chem. (2005) 77:259–65. doi: 10.1021/ac048788u, PMID: 15623304

[64] Pham MD Wen TC Li HC Hsieh PH Chen YR Chang HC. Streamlined membrane proteome preparation for shotgun proteomics analysis with triton X100 cloud point extraction and nanodiamond solid phase extraction. Materials. (2016) 9:385. doi: 10.3390/ma9050385, PMID: 28773508 PMC5503057

[65] Zhang Z Li D Ma X Li X Guo Z Liu Y. Carboxylated nanodiamond-mediated NH2-PLGA nanoparticle-encapsulated fig polysaccharides for strongly enhanced immune responses *in vitro* and in *vivo* . Int J Biol Macromol. (2020) 165:1331–45. doi: 10.1016/j.ijbiomac.2020.10.010, PMID: 33045302

[66] Ghoneum M Ghoneum A Gimzewski J. Nanodiamond and nanoplatinum liquid, DPV576, activates human monocyte-derived dendritic cells *in vitro* . Anticancer Res. (2010) 30:4075–9., PMID: 21036722

[67] Malina T Kaur J Martin S Gallud A Katayama S Gazzi A. Nanodiamonds interact with primary human macrophages and dendritic cells evoking a vigorous interferon response. ACS Nano. (2025) 19:19057–79. doi: 10.1021/acsnano.4c18108, PMID: 40368637 PMC12120995

