# Supplementary figures and images for: A nanodiamond-formulated plant protein induces robust immunity against porcine epidemic diarrhea virus in piglets
Source: Front Immunol. 2025 Sep 19;16:1674222. doi: 10.3389/fimmu.2025.1674222 (PMC12491242; doi:10.3389/fimmu.2025.1674222)

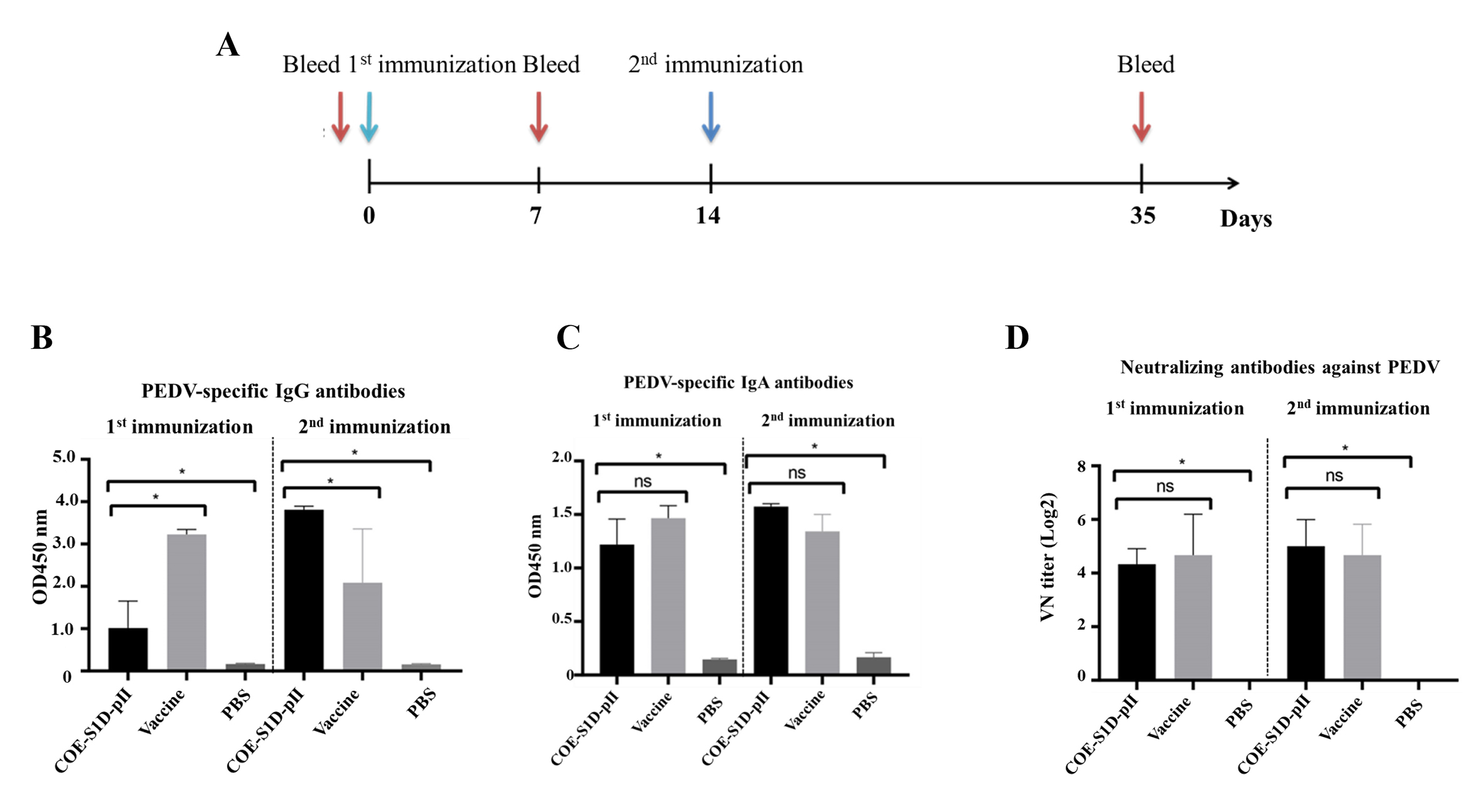

Supplement: Supplementary Figure 1 — Humoral immune responses in piglets induced by COE-S1D-pII protein and a commercial vaccine. (A). Piglet immunization scheme experiment. COE-S1D-pII protein (50 µg/dose) mixed Emulsigen®-D adjuvant (MVP) or inactivated vaccine containing the PEDV AJ1102 + TGEV WH-1 strains (Keqian Biology, Wuhan Keqian Biology, China) or PBS mixed Emulsigen®-D adjuvant (MVP) was immunized in piglets. The red arrow and blue arrow show bleeding time and vaccination, respectively. (B). COE-S1D-specific IgG antibodies in piglet sera were evaluated by ELISA using SEC-purified COE-S1D-pII as antigen. Data are presented as mean ± Standard deviation (SD). *indicates a statistically significant difference (p< 0.05). (C). COE-S1D-specific IgA antibodies in piglet sera were evaluated by ELISA using SEC-purified COE-S1D-pII as antigen. Data are presented as mean ± Standard deviation (SD). *indicates a statistically significant difference (p< 0.05). (D). Neutralizing antibodies in piglet sera were analyzed by the virus-neutralizing assay. PEDV SM98 (103 TCID50/0.1 mL) was used for the assay. A VN titer equal to or greater than 8 was considered indicative of a positive presence of neutralizing antibodies against PEDV. [file Image1.jpeg]
